# Changes in low-level neural properties underlie age-dependent visual decision making

**DOI:** 10.1038/s41598-018-27398-x

**Published:** 2018-07-17

**Authors:** Elahe Arani, Raymond van Ee, Richard van Wezel

**Affiliations:** 1 Biophysics Department, Donders Institute for Brain, Cognition and Behaviour, Radboud University, 6525AJ Nijmegen, The Netherlands; 2 Department of Brain and Cognition, Leuven University, BE-3000 Leuven, Belgium; 3 Department of Brain, Behavior and Cognition, Philips Research, Eindhoven, The Netherlands; 4 Biomedical Signal and Systems Group, MIRA Institute for Biomedical Technology and Technical Medicine, University of Twente, Enschede, The Netherlands

## Abstract

Aging typically slows down cognitive processes, specifically those related to perceptual decisions. However, the neurobiological mechanisms underlying these age-associated changes are still elusive. To address this, we studied the effect of aging on both perceptual and binocular rivalry in various presentation conditions. Two age groups of participants reported their spontaneous percept switches during continuous presentation and percept choices during intermittent presentation. We find no significant age effect on the mean and cumulative frequencies of percept switch durations under continuous presentation. However, the data show a significant age effect on coefficient of variation, ratio of standard deviation to mean of percept durations. Our results also reveal that the alternation rate for percept choices significantly declines at an older age under intermittent presentation. The latter effect is even more pronounced at shorter inter-stimulus durations. These results together with the predictions of existing neural models for bistable perception imply that age-dependency of visual perceptual decisions is caused by shifts in neural adaptation and noise, not by a change in inhibition strength. Thus, variation in the low-level neural properties, adaptation and noise, cause age-dependent properties in visual perceptual decisions.

## Introduction

The strong growth of the aging population will be accompanied by increasing numbers of people suffering from age-related dysfunctional cognitive processes. Understanding the underlying mechanisms of age-related cognitive decline is therefore essential in our aging society^1^. Earlier studies have demonstrated slower cognitive processes in older adults^2^. An important fundamental cognitive process that changes with age is visual perceptual decision making^3^. However, the underlying neural mechanisms remain unclear. We approach this problem by studying age-dependent perception of bistable visual stimuli, because much is known about the underlying mechanisms of bistable perception^4^. Bistable perception is a phenomenon in which perception switches between two rivaling interpretations of an unchanging stimulus^5,6^. There are two well-known forms of bistable perception: perceptual rivalry and binocular rivalry. Perceptual rivalry of ambiguous figures, such as the Necker cube, leads to alternations between two possible pictorial interpretations, whereas binocular rivalry involves perceptual alternations between two distinct images in the two eyes. Several neurobiological factors play a key role in perceptual and binocular rivalry: neural noise^7,8^, adaptation of the neural populations coding for the two different percepts^9,10^, and cross-inhibition of two competing neural populations^11,12^. Previously, it has been shown that older adults compared to young adults experience longer switch durations for perceptual rivalry^13–15^ as well as for binocular rivalry^16,17^. However, the role of these neural factors are still unclear.

It is well established that the minimal neurobiological factors to explain underlying mechanisms of bistable visual perception are cross-inhibition, adaptation (and/or short-term synaptic depression) and noise^18–25^. If cross-inhibition would be the only factor, the first perceived percept should always remain dominant, but this is not happening in practice^26,27^. To explain percept switches, spike frequency adaptation (SFA) is added in many models for bistable visual perception. Spike frequency adaptation leads to a reduction in the firing frequency of neural responses after an initial increase^28^, and therefore, the neural population for the other percept can become dominant. In other models, noise causes the switches between alternative percepts^29^. Other studies suggest that the serial correlations of percept durations can be explained specifically by noise in adaptation, but not by noise in cross-inhibition^30^.

Since it is difficult to study the role of cross-inhibition, adaptation and noise separately, we concentrate on varying one of the factors by using different experimental conditions: continuous presentation and intermittent presentation of visual stimuli. During continuous presentation, the bistable stimulus is constantly presented and a participant is instructed to report percept switches. During intermittent presentation, the bistable stimulus is presented for short presentation durations interrupted by an inter-stimulus interval with no stimulus presented and a participant is instructed to report the percept choice for each stimulus presentation. It has been shown that the percept choices are fundamentally different form percept switches^31–34^, and depend crucially on a formerly neglected, near-threshold interaction effect between the local adaptation mechanism and a small neural baseline^18^. A neural model showed that percept choices intervene depending on the combination of bias in activation and adaptation states of both populations in the brief time between onset and the perception, whereas percept switches take place when a slow, noisy accumulation of adaptation in the dominant percept gradually reduces its stability until any small noise can trigger a fast switch into opposite percept^35^. It has not only been shown that intermittent presentation recruits higher processing regions compared to continuous presentation^36^, but it also elicits no alternations at longer inter-stimulus intervals (>1 sec), which is often referred to as perceptual memory^37,38^. The differences between continuous and intermittent presentation predict different effects for changes in cross-inhibition, adaptation, and noise.

**Figure 1:**
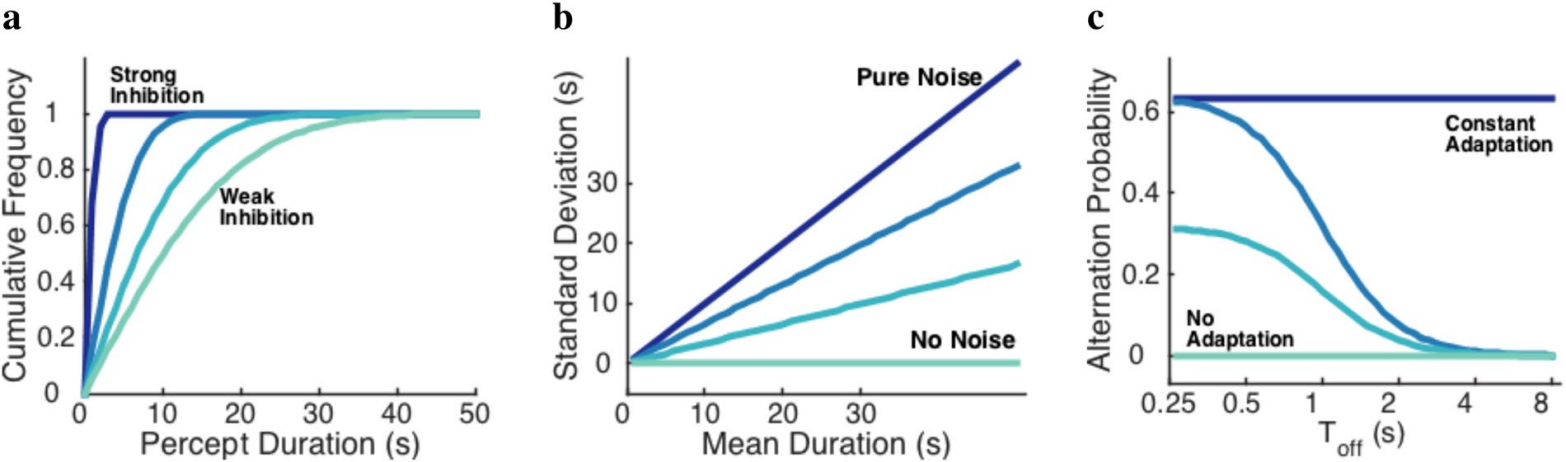
Role of neural factors in visual perceptual decisions. Predictions based on neural models of bistability by varying cross-inhibition, adaptation, and noise level. (**a**) Cumulative frequency of percept durations with weak (light blue) to strong inhibition (dark blue), (**b**) coefficient of variation in percept switch conditions with no noise (pure adaptation; light blue) to pure noise (no adaptation; dark blue), and (**c**) alternation rate for different inter-stimulus intervals (Toff) of intermittently presented bistable stimuli. The dark blue color indicates constant adaptation that is not changing during the inter-stimulus interval. The lighter lines indicate less adaptation up to no adaptation at all.

First, we focus on the inhibition factor by studying the cumulative frequency of mean percept durations in percept switch conditions. According to existing neural models, more inhibition leads to slower perceptual dynamics and hence longer percept durations^39,40^. The Cumulative frequency of percept durations should thus look like Fig. 1a. Second, we study the role of noise by analyzing the coefficient of variation (CV) of the percept durations in the percept switch conditions^24^. CV is defined by the standard deviation of percept durations divided by the mean, i.e. the slope of the linear regression line. Without noise (only adaptation), the switches would be completely deterministic, so there is no fluctuation in percept duration over time and the coefficient of variation is equal to zero. However, with only noise (no adaptation), the percept durations would have an exponential distribution, and therefore the coefficient of variation approaches one (see Fig. 1b). Finally, we focus on the adaptation factor by investigating the alternation rate in the percept choice condition^18,41^. It is also known that neural adaptation decreases with aging and slower adaptation causes a neuronal population to inhibit itself slower, therefore, leading to slower perceptual dynamics^42^ (Fig. 1c). Our study could thus in principle allow us to assess the relative contributions from the different neural factors to age-dependent perceptual decisions.

**Figure 2:**
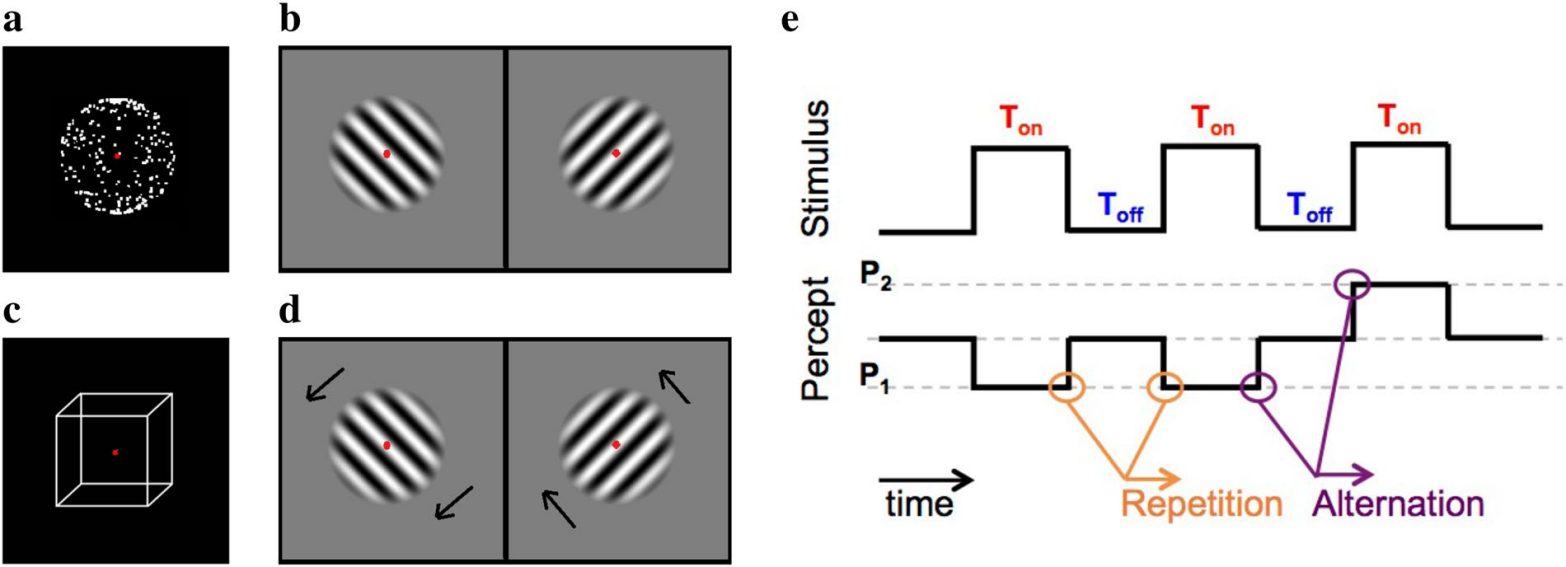
Visual stimuli and experimental procedure. Stimuli: (**a**) structure from motion (SFM) sphere, (**b**) binocular rivalry of static gratings presented in the left and right eye, (**c**) Necker cube, and (**d**) binocular rivalry of drifting gratings presented in the left and right eye. (**e**) Experimental procedure for the percept choice condition: stimuli were presented intermittently with various inter-stimulus durations (upper panel) for one-second stimuli duration. During each stimulus presentation, the participants reported the perceived percept (lower panel). Two subsequent similar percepts are defined as a repetition, and two subsequent different percepts are defined as an alternation.

Even though similar dynamics of perceptual and binocular rivalry have been used as evidence for similar underlying neural mechanisms, some studies have shown that binocular rivalry, compared to perceptual rivalry, involves a more automatic and stimulus-driven form of visual competition occurring at lower levels of the visual pathway^43–46^. We, therefore, employed four different stimuli divided into two experiments, so that there was one perceptual and one binocular stimulus in each experiment (Fig. 2). Experiment 1 (Exp1) consisted of an ambiguously moving structure from motion (SFM) sphere (moving perceptual rivalry) and static gratings presented in two eyes with orthogonal orientations (static binocular rivalry) and Exp2 consisted of a Necker cube (static perceptual rivalry) and drifting gratings with orthogonal orientations for the two eyes (moving binocular rivalry). Forty-two and thirty-one participants performed the experiments in a random order in Exp1 and Exp2, respectively. We calculated mean and CV of percept duration in percept switch conditions, and the alternation rate in percept choice conditions. We also computed the two eyes’ dominance biases relative to one another during binocular rivalry stimuli (chronic bias, see below). In our analyses, the participants were divided into a young (<31) and an old (>45) age-group.

## Results

**Figure 3:**
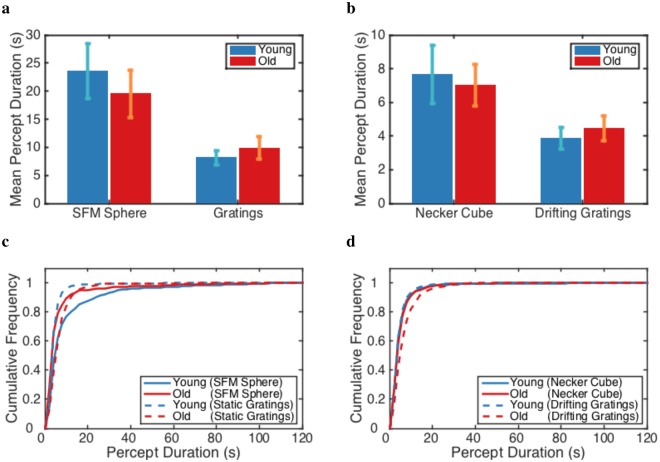
Mean and cumulative frequency of percept durations for perceptual and binocular rivalry in percept switch condition is not consistently and significantly different for the two age groups. (**a**,**b**) The mean perception durations, (**c**,**d**) the cumulative frequency of percept durations for both age groups (young in blue and old in red) in the continuous presentation for participants in Exp1 and Exp2, respectively. Error bars represent ±1 SEM.

For continuous presentation, our data did not show a difference between the age groups in mean percept duration for any of the four stimuli (with *p* = 0.53, *p* = 0.48, *p* = 0.77, and *p* = 0.53 for SFM sphere, static gratings, Necker cube, and drifting gratings, respectively) (Fig. 3a,b). For young adults, the mean percept duration for perceptual rivalry appeared to be slightly larger than for binocular rivalry. In addition, we found no difference in shape (*a*) and scale (*b*) parameters of Gamma distributions fitted to the percept duration for age groups (Avg. (*a*,*b*) = (6.50, 10.60), (4.29, 4.91), (2.28, 6.61), and (3.14, 3.58) for young group, and Avg. (*a*,*b*) = (2.71, 11.40), (4.04, 3.66), (3.51, 2.73), and (2.99, 4.03) for old group in SFM sphere, static gratings, Necker cube, and drifting gratings, respectively). Importantly, there was no consistent age effect on the cumulative frequencies for our participants in Exp1 and there was no significant age effect for participants in Exp2 (Fig. 3c,d).

**Figure 4:**
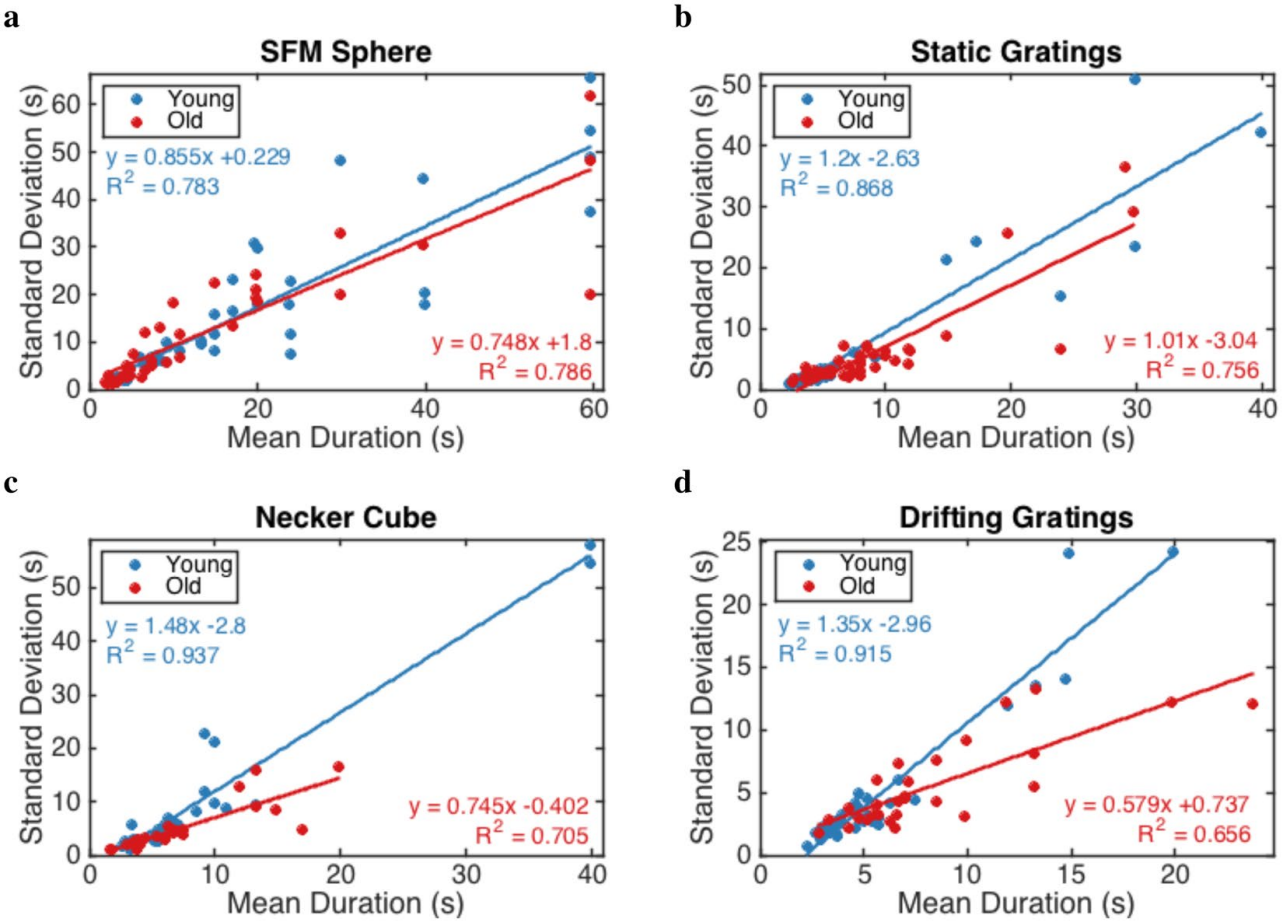
The coefficient of variation (CV) reveals a consistent aging effect on the mean percept duration in the switch percept condition for perceptual and binocular rivalry. CV is defined as the standard deviation of percept durations divided by the mean of percept durations, which is equal to the slope of linear regression lines. The effect of age on CV for all stimulus is significant, with *p* < 0.05 for static gratings, and *p* < 0.0001 for the other stimuli.

We demonstrate the influence of noise and adaptation on switches by calculating the coefficient of variation (CV), which is a measure of the balance between noise and adaptation (Fig. 4). The data show a larger CV for younger compared to older adults, which indicates stronger effect of noise for the young age group. To determine whether the CV of two groups differ significantly, we used a statistical approach to obtain the p-value from F ratio of group fits and the fit of pooled data (see Methods). We found a significant effect of age on CV for all stimuli (*p* < 0.05 for static gratings, and *p* < 0.0001 for the other stimuli). Our analysis of CV’s suggests that the age-related changes in dynamics of percept switches are at least partly due to changes in the strength of noise and adaptation.

**Figure 5:**
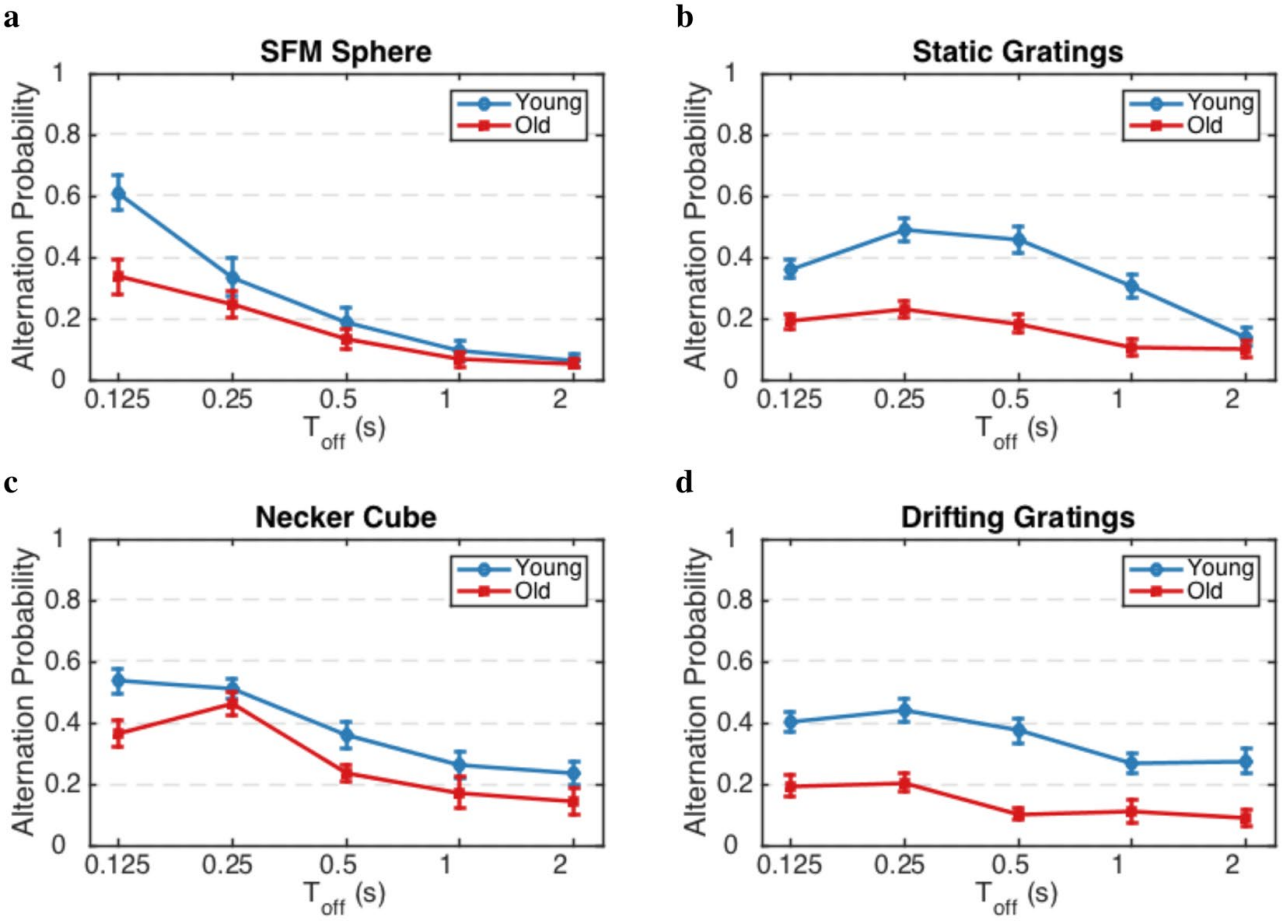
Alternation probability in percept choice condition declines with age and at longer Toff. Average alternation probability for all participants divided into two young (blue) and old (red) age groups as a function of Toff (Ton = 1 s in all conditions). Error bars represent $\pm 1$ SEM. A profound effect of age is evident in all four conditions.

Having discussed the dynamics of percept switches, we now turn to the dynamics of percept choices. To examine the effects of age on these dynamics, we calculated the alternation rate for various inter-stimulus durations (Toff) and different age groups for both perceptual and binocular rivalry (Fig. 5). Stimuli were presented intermittently with various Toff for one-second stimulus presentation (Ton), which during each Ton, the participants reported the perceived percept. Two subsequent different percepts are defined as an alternation. We used a two-way ANOVA to test the influence of age group and Toff on alternation probability. For the SFM sphere (Fig. 5a), both the effects of age groups (*p* < 0.0001) and Toff (*p* < 0.0001) were strong, the significant interaction was evident between the two factors (*p* < 0.001). First and foremost, note that the alternation probability for the SFM sphere decreases with Toff. Second, the alternation probability for the two age groups converge. The data for the SFM sphere point to a clear role of adaptation plus noise (as opposed to inhibition, thus supporting the results of the aforementioned continuous presentation data): the change of alternation probability over time reveals a role of adaptation with time because, at larger Toff, the adaptation effect washes out. T-tests shows a significant age effect at shorter Toffs (125 and 250 ms) for all four stimuli; p-value = 0.01, 1.6e-6, 0.008, and 4e-5 for SFM sphere, static gratings, Necker cube, and drifting gratings, respectively. Since the more pronounced age-related differences in alternation probability is at the shorter Toff, we conclude that the adaptation component plays a relatively larger role in the effect of age in the dynamics of the perceptual decisions (supporting results on CV; Fig. 4). Results for the static gratings were similar. A two-way ANOVA disclosed strong effects of age groups (*p* < 0.0001), Toff (*p* < 0.0001) and the presence of a significant interaction between the two (*p* < 0.005). Results for the Necker cube and drifting gratings are slightly different at longer Toff. A two-way ANOVA revealed strong effects of age groups (*p* < 0.0001) and Toff (*p* < 0.0001), whereas there were no significant interactions between the two factors ($p\,=\,0.50$ and $p\,=\,0.61$ for Necker cube and drifting gratings, respectively). Convergence might thus take place at a longer Toff. In summary, the young age group exhibited larger alternation probability than the old age group. At longer Toff, the alternation probability declined and at the shorter Toff, the difference in alternation probability between age groups was more pronounced.

**Figure 6:**
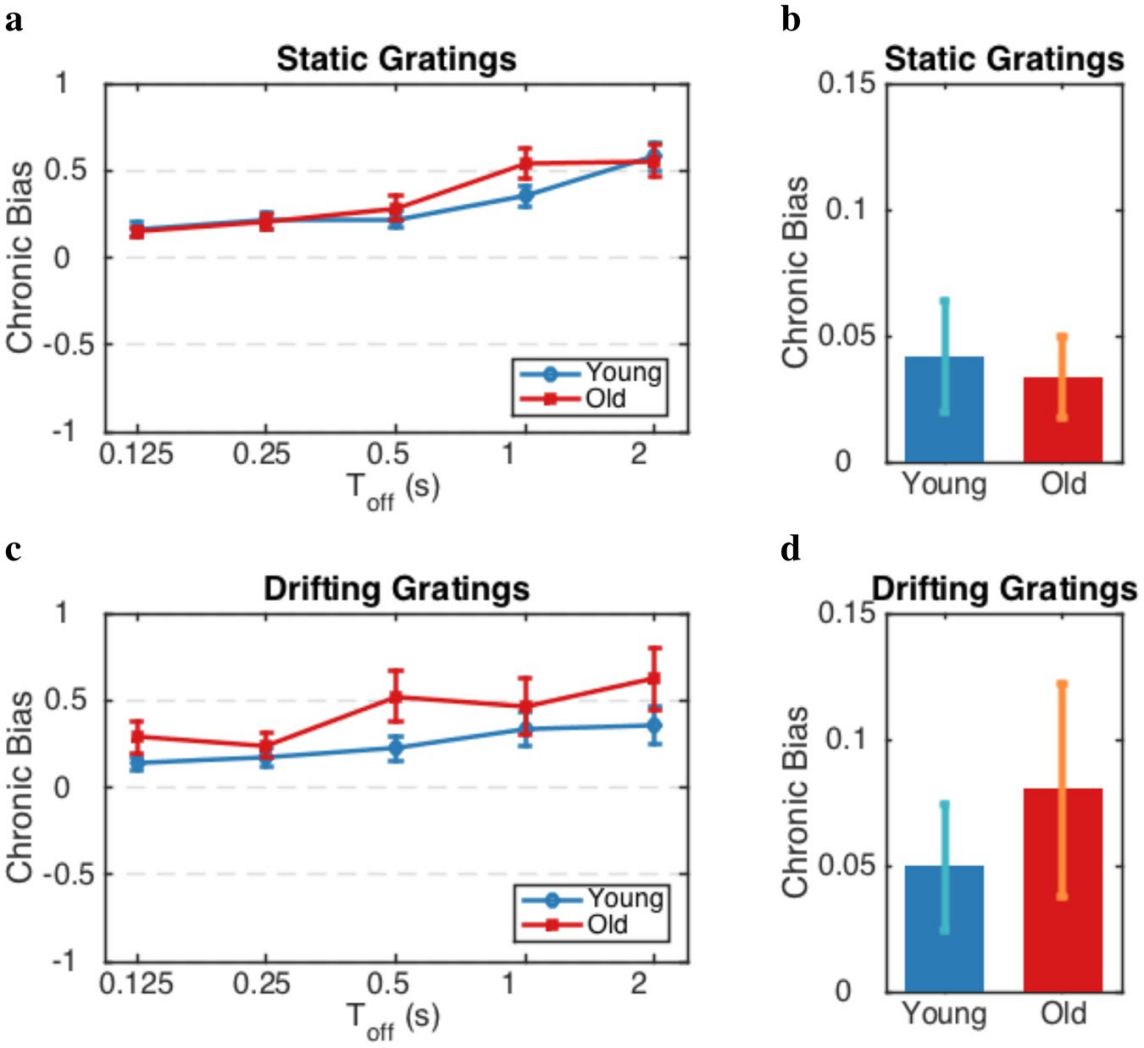
Aging does not affect the chronic bias. Chronic biases for participants of (**a,b**) Exp.1 based on static gratings and (**c,d**) Exp.2 based on drifting gratings. (**a,c**) are for the percept choice conditions and (**b,d**) are for the percept switch conditions. Error bars represent $\pm 1$ SEM. chronic bias shows a long-term, intrinsic bias for a given perceptual state. The data in these experiments do not reveal an age-related difference in chronic bias between two age groups.

To determine whether the difference in the dynamics of perceptual decision is caused by eye deterioration due to aging, we computed the chronic bias with the data of binocular rivalry (see Methods) in both experiments for both age groups (Fig. 6). Chronic bias indicates one eye’s bias in dominance irrespective of perceptual history which shows a long-term, intrinsic bias for a given perceptual state^47^. A two-way ANOVA revealed a significant main effect of the Toff ($p < 0.05$) for both experiments. The interaction between both age and Toff was not significant ($p\simeq 0.96$ and $p\simeq 0.67$ for Ex.1 and Ex.2, respectively), indicating that age does not have a significant effect on chronic bias at different Toff conditions. Also, an ANOVA did not show any significant age effect on the average chronic bias over all participants in each age group during the switch percept conditions ($p\,=\,0.88$ and $p\,=\,0.21$ for the participant in Ex.1 and Ex.2, respectively). Here, we showed that the decrease in the alternation rate with aging is highly unlikely to be caused by eye deterioration.

## Discussion

To investigate the effect of age on perceptual decisions, we examined percept switches and percept choices for different age groups, and for different visual stimuli. While the effect of age on mean percept duration and cumulative frequency of percept switches during continuous stimulation was insignificant for the visual stimuli that we used, the data showed that the alternation rate of percept choices under intermittent stimulus presentation and the coefficient of variation under continuous presentation decreased with age. Based upon our findings and the model-based predictions in Fig. 1, we argue that reduced neural adaptation and noise cause the age-dependency in perceptual decision. We also argue that this age-dependency cannot be simply explained by differences in eye deterioration, especially for the older age group.

We interpret our results that age-dependency of visual decisions is not correlated with changes in inhibition between two competing neural populations representing two possible percepts. However, it has previously been hypothesized that slower dynamics for older people is caused by increased inhibitory activity in visual cortex^39^, but no arguments were provided to exclude the role of adaptation and/or noise. There are observations using post-mortem brains of an age-related decline in gamma-aminobutyric acid (GABA) transporters in human cortex^48^. However, this was only found in frontal cortex and it is known that GABA transporter densities are highly variable in human cortex^49^. Further studies on GABA transporters in visual cortex in different age groups are needed to confirm our conclusions.

We found that the ratio of standard deviation to mean (CV) of percept durations significantly decreases by aging. Additionally, alternation rate decreased with age and this decline is even stronger at the shorter inter-stimulus durations. These two results demonstrate the age-dependent variation in adaptation and noise. In line with our findings, it has been shown that aging might reduce the variability of activity across larger areas of cortex^50^ and that increased readiness of adaptation is accompanied by faster alternation rates for children in binocular rivalry^42^. Further non-invasive neural measurement, e.g. EEG, might be needed to address the age-related neural noise.

We studied visual decision making with both binocular and perceptual rivalry. These two perceptual decision making tasks have been studied extensively, also in relation to aging. We find similar effects of aging on both perceptual and binocular rivalry, which is evidence for similar underlying neural mechanisms. However, some studies have shown that binocular rivalry, compared to perceptual rivalry, involves a more automatic and stimulus-driven form of visual competition occurring at lower levels of the visual pathway^43–46^. A seminal extensive study with 252 participants ranging from 5-year-old to 95-year-old showed that the number of switches during continuous presentation in perceptual rivalry are about equal for young and old age groups^13^. While some more recent studies reported that younger adults exhibit a larger number of switches than older adults during continuous presentation of visual rivalry^15,17,42^, our results are consistent with the older seminal study^13^.

Our measurements rely only on subjective responses, and does not include attentional control^26,51,52^ or eye-movement control^53,54^. Therefore, effects of eye movements and/or attention on our results can not be excluded. An effect of eye movements and attentional control would imply, however, that the younger group has different eye movement or attentional strategies to perform the task than the older group. From our measurements or the literature, we have no evidence that this might hold.

We showed for perceptual decisions that cognitive decline is the result of low-level changes of neural activity (i.e., adaptation and noise). These results might be highly relevant for our aging society, because it could provide a more mechanistic account of aging and inspiration for new research directions. We suggest to repeat our analyses across different tasks and different stimuli, accompanying with a (non-invasive) neural activity measuring technique to confirm our neurobiological hypothesis and investigate it in further detail. Our interpretations of the results might be debatable and one can take it as a further starting point for new studies to falsify our hypotheses.

## Methods

### Visual Stimulus

In this study, we used four different bistable stimuli: the rotating structure from motion (SFM) sphere, Necker cube, and binocular rivalry of static or moving sinusoidal gratings.

An ambiguously rotating structure-from-motion (SFM) sphere was composed of two transparent layers of random white dot patterns on a black background moving in opposite directions with a sinusoidal speed profile (Fig. 2a). Due to structure-from-motion effects, these moving dots created the vivid impression of a three-dimensional rotating sphere^55,56^. The dots were 2.8 arcmin and moved with a sinusoidal speed profile with a peak angular speed of 60 degrees/second. The luminance of the white dots was 21.7 $cd/{m}^{2}$ and background luminance was 0.13 $cd/{m}^{2}$; the dot density was 40 dots per squared visual degree. The dot lifetime was infinite, but at the start of each stimulus presentation, the dots were randomly positioned to prevent tracking individual dots over stimulus presentations.

Two orthogonally oriented gratings presented binocularly had spatial frequencies of 1.75 cycles/degree and were tilted clockwise (right eye) and anti-clockwise (left eye) with an angle of 45 degrees, while the orientation remained the same for each eye throughout the whole experiment. A sinusoidal contrast function was applied for the formation of alternating white and black stripes. The gratings (Fig. 2b) were overlaid with a Gaussian mask ($\sigma =0.5$ degree) to blend in the edges of the gratings with the gray background. In the first experiment the gratings were stationary, and in the second experiment, the gratings were moving upward (right eye) and downward (left eye; Fig. 2d).

The Necker cube has been among the most studied bistable ambiguous stimuli^44,57,58^ which is alternately perceived as if it was viewed from above and from below (Fig. 2c). The luminance of the white lines was 21.7 $cd/{m}^{2}$ and background luminance was 0.13 $cd/{m}^{2}$.

The stimuli had sizes of 2.4 degrees in diameter with a red fixation square ($4.2\,\times \,4.2$ arcmin) in its center. They were presented on a 22 inch CRT screen (LaCie Electron 22 blue IV) with a resolution of $1600\,\times \,1200$ pixels (or a size of $390\,\times \,295$ millimeters) and a refresh rate of 100 Hz. In a completely dark room, participants viewed the stimuli through a mirror stereoscope, at a distance of 100 cm from the screen. They reported their visual percept by using the left or right arrow of the keyboard. To support correct binocular fusion of the images, in the first experiment, rivalry stimuli were accompanied by a visual reference in periphery presented to both eyes, whereas, in the second experiment, the checkerboard was replaced by four surrounding cross-hairs (with a size of $0.975\,\times \,0.062$ degrees) that were added at a distance of 1.45 degree. The experiments were programmed in MATLAB (The MathWorks Inc. 2014b) using PsychToolbox_3.

### Experimental Protocol

For all four stimuli, the experiments contained a percept switch (continuous presentation) and a percept choice (intermittent presentation) condition. To inform the participants of the upcoming condition, instructions were presented on the screen before the start of each block. In the percept switch blocks, participants reported their percept in the beginning and later when their percept switched to the other percept. In the percept choice blocks, participants had to report their first percept after each stimulus onset (Fig. 2e). As it has been shown that the influence of stimulus duration (Ton) on alternation probabilities is quite small for these ambiguous stimuli^26^, we only varied the inter-stimulus duration (Toff = 125, 250, 500, 1000 and 2000 milliseconds) and used a fixed one-second stimulus duration. Together with the continuous presentation condition, this resulted in six different conditions each presented twice in pseudo-random order in blocks of two minutes duration for each stimulus. The total duration of each experiment, therefore, was $2\,\times \,24$ minutes. The participants were asked to always report the most dominant percept. Before the experimented started, participants performed a two minutes trial experiment and the experimenter checked whether they could perceive and report the percepts properly. The experimental protocol was approved by the Radboud Ethical committee (ECSW2016-2208-41).

### Participants

In both experiments, participants were recruited from the students, employees, retired persons of Radboud University and some local seniors. All volunteers had normal (or corrected to normal) vision (Table 1). Participants were asked to adjust the position of the dichoptic mirrors of the stereoscope to get an entirely aligned image. They passively watched the screen and reported their percept by pressing either the left or the right arrow on the keyboard, corresponding to the percept. Participants gave their informed consent and the experiments were in accordance with the Radboud University ethics and safety guidelines.

**Table 1:** Participants’ information.

	Female		Male		Young		Old	
	No.	Age	No.	Age	No.	Age	No.	Age
Stimuli: SFM sphere and static gratings								
Exp.1	20	34.6 ± 17.7	22	46.6 ± 19.8	21	22.7 ± 3.8	21	59.2 ± 8.4
Stimuli: Necker cube and drifting gratings								
Exp.2	13	33.2 ± 18.0	18	42.0 ± 19.4	17	21.8 ± 2.4	14	58.4 ± 6.2

### Data Analysis

For the percept switch conditions, the average percept duration was calculated in each block. A key press identical to the previous one was considered as a continuation of the percept. The last registered key-response within a block was ignored because it was truncated by the end of the trial, as it contains no reliable information about the duration of the percept. We then fitted a Gamma distribution, because shape and scale parameters can be informative about the underlying neural process^59^. We finally used the mean percept duration to create the cumulative percept duration histograms of both age groups.

We determined the standard deviation of percept durations versus the mean percept durations for each participant. We fitted a linear regression to each group where the slopes represent the corresponding coefficients of variation (CV). To determine whether CV between two age groups differ significantly, we used a generalized F-test to compare two different sets of data^60^. First, the two groups are analyzed separately, with residual sum of squares $S{S}_{group}=S{S}_{old}+S{S}_{young}$ and the number of degrees freedom $d{f}_{group}=d{f}_{old}+d{f}_{young}$. Second, the pooled data is analyzed and yields values $S{S}_{pool}$ and $d{f}_{pool}$. Then, the statistical significance of the improvement is determined from the F ratio calculated as

1 $$F=\frac{(S{S}_{pool}-S{S}_{group})/(d{f}_{pool}-d{f}_{group})}{S{S}_{group}/d{f}_{group}}.$$

To interpret the meaning of this F value, a statistical table is used to convert to a p-value, in which the numerator and the denominator have $d{f}_{pooled}-d{f}_{grouped}$ and $d{f}_{grouped}$ degrees of freedom, respectively. A small p-value (corresponded to a large F value) indicates that the separate fit is much better than the pooled fit.

We also analyzed data based on a perceptual index (chronic bias), which is defined as the property of single percepts^47^. The chronic bias is defined as the difference between the probability in predominance of one eye’s stimulus and the other eye’s stimulus in determining the perceived stimulus. In other words, the chronic bias indicates one eye’s bias irrespective of perceptual history. It ranges from −1 to +1 and is zero when there is no bias. For the continuous presentation, the duration of the right-eye percept divided by the total percept duration is defined as the probability of the right-eye.

For the percept choice conditions, two subsequent stimulus presentations with different responses are defined as an alternation (Fig. 3e), and the fraction of the total number of alternations (alternation probability) was calculated. Participants were instructed to respond only once during the one-second stimulus duration. If multiple key-presses were given, the first response was taken as the response and later ones were excluded. Trials in which the participant failed to respond were also excluded, along with their preceding and subsequent trials.

## Electronic supplementary material

LaTeX Supplementary File

LaTeX Supplementary File

LaTeX Supplementary File

LaTeX Supplementary File

LaTeX Supplementary File

LaTeX Supplementary File

LaTeX Supplementary File

LaTeX Supplementary File

LaTeX Supplementary File

LaTeX Supplementary File

LaTeX Supplementary File

LaTeX Supplementary File

LaTeX Supplementary File

LaTeX Supplementary File

LaTeX Supplementary File

LaTeX Supplementary File

LaTeX Supplementary File

LaTeX Supplementary File

LaTeX Supplementary File

LaTeX Supplementary File

LaTeX Supplementary File

LaTeX Supplementary File

LaTeX Supplementary File

LaTeX Supplementary File

## References

[1] Deary IJ (2009). Age-associated cognitive decline. British medical bulletin.

[2] Salthouse TA (1996). The processing-speed theory of adult age differences in cognition. Psychological review.

[3] Glisky, E. L. Changes in cognitive function in human aging. *Brain aging: models, methods, and mechanisms* 3–20 (2007).

[4] Klink PC, van Wezel RJ, van Ee R (2013). The future of binocular rivalry research. The Constitution of Visual Consciousness: Lessons from Binocular Rivalry.

[5] Leopold DA, Logothetis NK (1999). Multistable phenomena: changing views in perception. Trends in cognitive sciences.

[6] Blake R, Logothetis NK (2002). Visual competition. Nature Reviews Neuroscience.

[7] Brascamp JW, Van ER, Noest AJ, Jacobs RH, van den Berg AV (2006). The time course of binocular rivalry reveals a fundamental role of noise. Journal of vision.

[8] Kim Y-J, Grabowecky M, Suzuki S (2006). Stochastic resonance in binocular rivalry. Vision research.

[9] Nawrot M, Blake R (1989). Neural integration of information specifying structure from stereopsis and motion. Science.

[10] Kang M-S, Blake R (2010). What causes alternations in dominance during binocular rivalry?. Attention, Perception, & Psychophysics.

[11] Blake R, Westendorf D, Fox R (1990). Temporal perturbations of binocular rivalry. Attention, Perception, & Psychophysics.

[12] Alais D, Cass J, O’Shea RP, Blake R (2010). Visual sensitivity underlying changes in visual consciousness. Current biology.

[13] Holt GL, Matson JL (1976). The effects of age on perceptual changes using two new perspectives of the necker cube. Bulletin of the Psychonomic Society.

[14] Beer J, Beer J, Markley RP, Camp CJ (1989). Age and living conditions as related to perceptions of ambiguous figures. Psychological reports.

[15] Aydin S, Strang NC, Manahilov V (2013). Age-related deficits in attentional control of perceptual rivalry. Vision research.

[16] Jalavisto E (1964). The phenomenon of retinal rivalry in the aged. Gerontology.

[17] Ukai K, Ando H, Kuze J (2003). Binocular rivalry alternation rate declines with age. Perceptual and motor skills.

[18] Noest A, Van ER, Nijs M, Van Wezel R (2007). Percept-choice sequences driven by interrupted ambiguous stimuli: a low-level neural model. Journal of vision.

[19] Lehky SR (1988). An astable multivibrator model of binocular rivalry. Perception.

[20] Laing CR, Chow CC (2002). A spiking neuron model for binocular rivalry. Journal of computational neuroscience.

[21] Wilson HR (2003). Computational evidence for a rivalry hierarchy in vision. Proceedings of the National Academy of Sciences.

[22] Wilson HR (2007). Minimal physiological conditions for binocular rivalry and rivalry memory. Vision research.

[23] Shpiro A, Curtu R, Rinzel J, Rubin N (2007). Dynamical characteristics common to neuronal competition models. Journal of neurophysiology.

[24] Shpiro A, Moreno-Bote R, Rubin N, Rinzel J (2009). Balance between noise and adaptation in competition models of perceptual bistability. Journal of computational neuroscience.

[25] Vattikuti S (2016). Canonical cortical circuit model explains rivalry, intermittent rivalry, and rivalry memory. PLoS computational biology.

[26] Klink P (2008). Early interactions between neuronal adaptation and voluntary control determine perceptual choices in bistable vision. Journal of vision.

[27] Logothetis NK, Leopold DA, Sheinberg DL (1996). What is rivalling during binocular rivalry?. Nature.

[28] Abbott LF, Varela J, Sen K, Nelson S (1997). Synaptic depression and cortical gain control. Science.

[29] Moreno-Bote R, Rinzel J, Rubin N (2007). Noise-induced alternations in an attractor network model of perceptual bistability. Journal of neurophysiology.

[30] van Ee R (2009). Stochastic variations in sensory awareness are driven by noisy neuronal adaptation: evidence from serial correlations in perceptual bistability. JOSA A.

[31] Sterzer P, Rees G (2008). A neural basis for percept stabilization in binocular rivalry. Journal of cognitive neuroscience.

[32] Guckenheimer, J. & Holmes, P. J. *Nonlinear oscillations, dynamical systems, and bifurcations of vector fields*, vol. 42 (Springer Science & Business Media, 2013).

[33] Pastukhov A, Lissner A, Füllekrug J, Braun J (2014). Sensory memory of illusory depth in structure-from-motion. Attention, Perception, & Psychophysics.

[34] Sandberg K (2014). Distinct meg correlates of conscious experience, perceptual reversals and stabilization during binocular rivalry. Neuroimage.

[35] Noest AJ, van Wezel RJ (2012). Dynamics of temporally interleaved percept-choice sequences: Interaction via adaptation in shared neural populations. Journal of computational neuroscience.

[36] Wang M, Arteaga D, He J (2013). Brain mechanisms for simple perception and bistable perception. Proceedings of the National Academy of Sciences.

[37] Leopold DA, Wilke M, Maier A, Logothetis NK (2002). Stable perception of visually ambiguous patterns. Nature neuroscience.

[38] Maier A, Wilke M, Logothetis NK, Leopold DA (2003). Perception of temporally interleaved ambiguous patterns. Current Biology.

[39] Hoshino O (2013). Ambient gaba responsible for age-related changes in multistable perception. Neural computation.

[40] van Loon AM (2013). Gaba shapes the dynamics of bistable perception. Current Biology.

[41] De Jong MC, Knapen T, Van Ee R (2012). Opposite influence of perceptual memory on initial and prolonged perception of sensory ambiguity. PLoS One.

[42] Hudak, M. *et al*. Increased readiness for adaptation and faster alternation rates under binocular rivalry in children. *Frontiers in human neuroscience* **5** (2011). 10.3389/fnhum.2011.00128 PMC3208241 22069386

[43] Tong F, Engel SA (2001). Interocular rivalry revealed in the human cortical blind-spot representation. Nature.

[44] Meng M, Tong F (2004). Can attention selectively bias bistable perception? differences between binocular rivalry and ambiguous figures. Journal of vision.

[45] Tong F, Meng M, Blake R (2006). Neural bases of binocular rivalry. Trends in cognitive sciences.

[46] Dowlati, E., Adams, S. E., Stiles, A. B. & Moran, R. J. Aging into perceptual control: a dynamic causal modeling for fmri study of bistable perception. *Frontiers in human neuroscience* **10** (2016). 10.3389/fnhum.2016.00141 PMC4814553 27064235

[47] Al-Dossari M, Blake R, Brascamp JW, Freeman AW (2015). Chronic and acute biases in perceptual stabilization. Journal of vision.

[48] Sundman-Eriksson I, Allard P (2006). Age-correlated decline in [3h] tiagabine binding to gat-1 in human frontal cortex. Aging clinical and experimental research.

[49] Inda M, Defelipe J, Munoz A (2006). The distribution of chandelier cell axon terminals that express the gaba plasma membrane transporter gat-1 in the human neocortex. Cerebral Cortex.

[50] Garrett DD, Kovacevic N, McIntosh AR, Grady CL (2011). The importance of being variable. Journal of Neuroscience.

[51] Lack, L. C. *Selective attention and the control of binocular rivalry*, vol. 11 (Mouton De Gruyter, 1978).

[52] Van ER, van Dam LC, Brouwer G (2005). Voluntary control and the dynamics of perceptual bi-stability. Vision research.

[53] Einhäuser W, Martin KA, König P (2004). Are switches in perception of the necker cube related to eye position?. European Journal of Neuroscience.

[54] van Dam LC, van Ee R (2006). Retinal image shifts, but not eye movements per se, cause alternations in awareness during binocular rivalry. Journal of Vision.

[55] Siegel R, Andersen R (1988). Perception of three-dimensional structure from motion in monkey and man. Nature.

[56] Andersen RA, Bradley DC (1998). Perception of three-dimensional structure from motion. Trends in cognitive sciences.

[57] George RW (1936). The significance of the fluctuations experienced in observing ambiguous figures and in binocular rivalry. The Journal of General Psychology.

[58] Toppino TC (2003). Reversible-figure perception: Mechanisms of intentional control. Attention, Perception, & Psychophysics.

[59] Van ER, Noest A, Brascamp J, van den Berg A (2006). Attentional control over either of the two competing percepts of ambiguous stimuli revealed by a two-parameter analysis: Means do not make the difference. Vision research.

[60] Motulsky HJ, Ransnas LA (1987). Fitting curves to data using nonlinear regression: a practical and nonmathematical review. The FASEB journal.

