# Supplementary figures and images for: Changes in low-level neural properties underlie age-dependent visual decision making
Source: Sci Rep. 2018 Jul 17;8:10789. doi: 10.1038/s41598-018-27398-x (PMC6050268; doi:10.1038/s41598-018-27398-x)

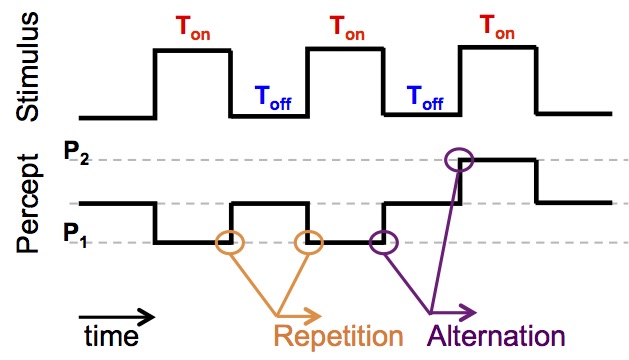

Supplement: Supplementary file 1 — LaTeX Supplementary File [file 41598_2018_27398_MOESM1_ESM.jpg]

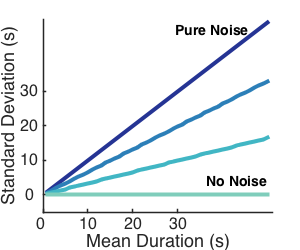

Supplement: Supplementary file 2 — LaTeX Supplementary File [file 41598_2018_27398_MOESM2_ESM.png]

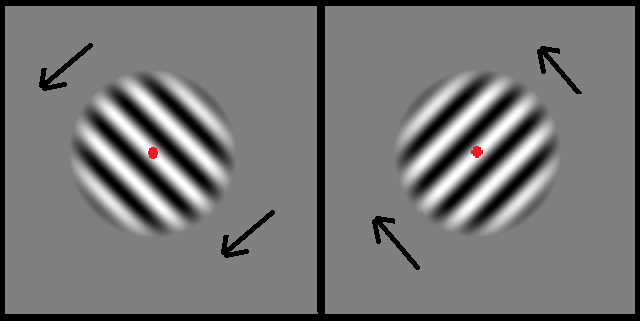

Supplement: Supplementary file 3 — LaTeX Supplementary File [file 41598_2018_27398_MOESM3_ESM.png]

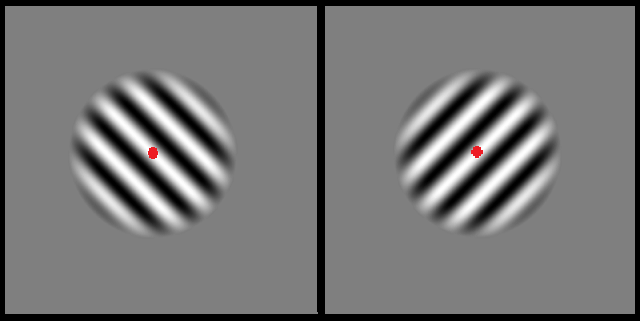

Supplement: Supplementary file 4 — LaTeX Supplementary File [file 41598_2018_27398_MOESM4_ESM.png]

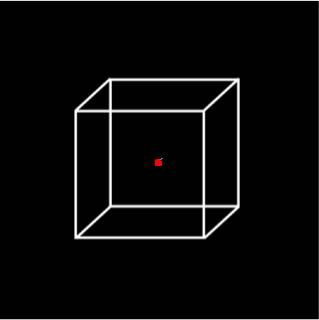

Supplement: Supplementary file 5 — LaTeX Supplementary File [file 41598_2018_27398_MOESM5_ESM.png]

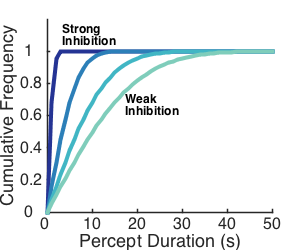

Supplement: Supplementary file 6 — LaTeX Supplementary File [file 41598_2018_27398_MOESM6_ESM.png]

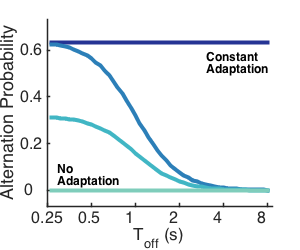

Supplement: Supplementary file 7 — LaTeX Supplementary File [file 41598_2018_27398_MOESM7_ESM.png]

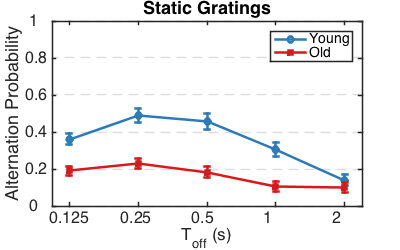

Supplement: Supplementary file 8 — LaTeX Supplementary File [file 41598_2018_27398_MOESM8_ESM.png]

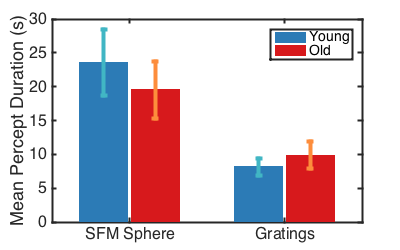

Supplement: Supplementary file 9 — LaTeX Supplementary File [file 41598_2018_27398_MOESM9_ESM.png]

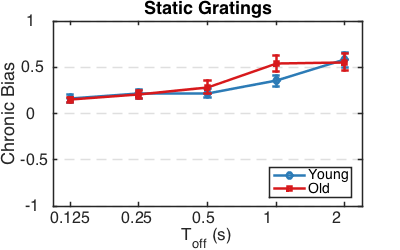

Supplement: Supplementary file 10 — LaTeX Supplementary File [file 41598_2018_27398_MOESM10_ESM.png]

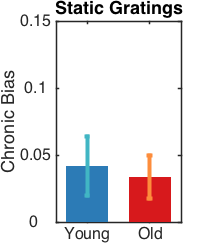

Supplement: Supplementary file 11 — LaTeX Supplementary File [file 41598_2018_27398_MOESM11_ESM.png]

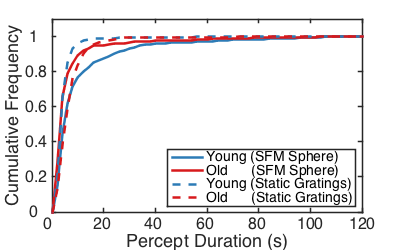

Supplement: Supplementary file 12 — LaTeX Supplementary File [file 41598_2018_27398_MOESM12_ESM.png]

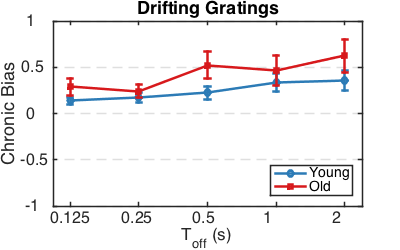

Supplement: Supplementary file 13 — LaTeX Supplementary File [file 41598_2018_27398_MOESM13_ESM.png]

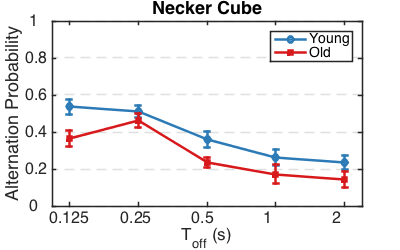

Supplement: Supplementary file 14 — LaTeX Supplementary File [file 41598_2018_27398_MOESM14_ESM.png]

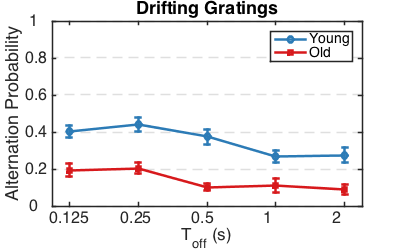

Supplement: Supplementary file 15 — LaTeX Supplementary File [file 41598_2018_27398_MOESM15_ESM.png]

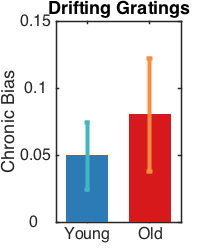

Supplement: Supplementary file 16 — LaTeX Supplementary File [file 41598_2018_27398_MOESM16_ESM.png]

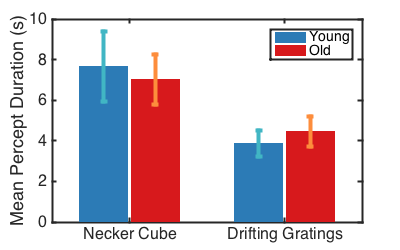

Supplement: Supplementary file 17 — LaTeX Supplementary File [file 41598_2018_27398_MOESM17_ESM.png]

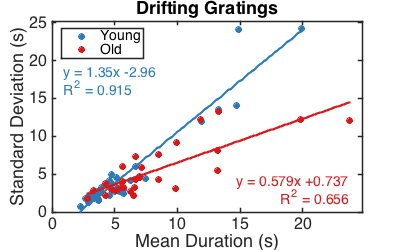

Supplement: Supplementary file 18 — LaTeX Supplementary File [file 41598_2018_27398_MOESM18_ESM.png]

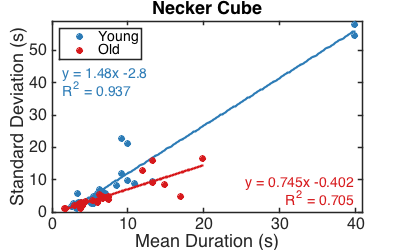

Supplement: Supplementary file 19 — LaTeX Supplementary File [file 41598_2018_27398_MOESM19_ESM.png]

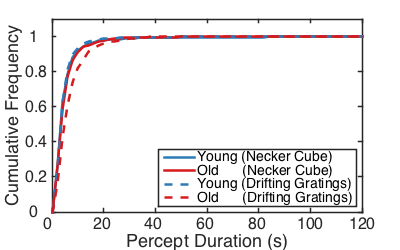

Supplement: Supplementary file 20 — LaTeX Supplementary File [file 41598_2018_27398_MOESM20_ESM.png]

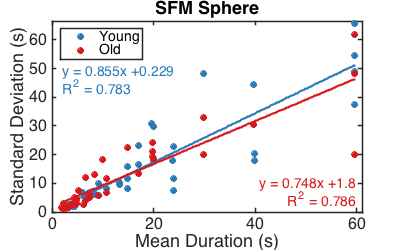

Supplement: Supplementary file 21 — LaTeX Supplementary File [file 41598_2018_27398_MOESM21_ESM.png]

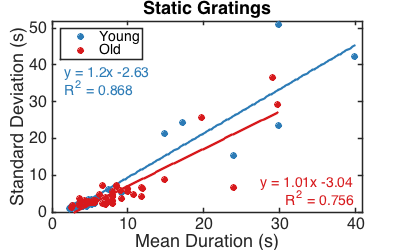

Supplement: Supplementary file 22 — LaTeX Supplementary File [file 41598_2018_27398_MOESM22_ESM.png]

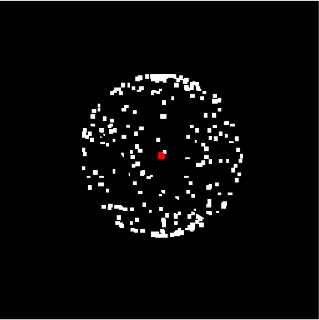

Supplement: Supplementary file 23 — LaTeX Supplementary File [file 41598_2018_27398_MOESM23_ESM.png]

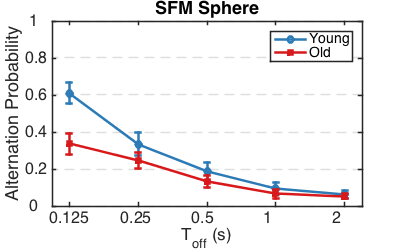

Supplement: Supplementary file 24 — LaTeX Supplementary File [file 41598_2018_27398_MOESM24_ESM.png]
